# Fine-grained evaluation of a domain-specific Q&A dataset to support trustworthy medical language models

**DOI:** 10.1007/s13755-026-00458-7

**Published:** 2026-04-24

**Authors:** Rafael da C. Fonseca, Ricardo A. Rios, Rodrigo Castaldoni, Adrielle A. Carvalho, Tiago J. S. Lopes, Caio L. B. Andrade, Braian V. G. Bispo, Laís R. Mota, Tatiane N. Rios

**Affiliations:** 1 Center for Advanced Analytics, Itaú Unibanco, Sao Paulo, Brazil; 2 Institute of Computing, Federal University of Bahia, Salvador, 40170-110 Brazil; 3 Nezu Life Sciences, 76149 Karlsruhe, Germany; 4 Institute of Health Sciences, Federal University of Bahia, Salvador, 40110-902 Brazil; 5 Laboratório de Genética Humana e Mutagênese, Salvador, 40110-902 Brazil

**Keywords:** Language models, Specialized domains, Q&A dataset, Generative models

## Abstract

The effective use of Large Language Models (LLMs) for generating coherent and informative content in specialized domains has largely been driven by the development of robust evaluation strategies. Based on this assumption, we introduce HemoQAL, a domain-specific question-and-answer (Q&A) dataset on hemophilia, derived from recent scientific publications and clinical guidelines. Our main contribution lies in a fine-grained evaluation of the quality of LLM-generated content. First, we carried out a human evaluation in which medical experts assessed the factual accuracy and educational value of the generated Q&A pairs. Second, we conducted a semantic similarity analysis to quantitatively evaluate the alignment between each Q&A pair and its original source material. These lightweight, scalable semantic metrics offer an efficient alternative to more resource-intensive human or LLM-based evaluation pipelines. Our findings show that integrating expert review with semantic similarity measures improves the reliability and trustworthiness of LLM-generated medical content, contributing to the development of dependable AI tools in health informatics.

## Introduction

Natural Language Processing (NLP) and language models have experienced an essential transformation in recent years, caused by the advent of Large Language Models (LLMs) [1, 2]. These models have demonstrated the ability to generate and extract information from human-like text. The versatility of LLMs allows performing a diverse array of natural language processing tasks, such as providing good responses in Question Answering (Q&A), generating coherent and contextually relevant text, facilitating translation between languages, summarizing long texts, performing sentiment analysis on texts, predicting the next word or sequence of words in a sentence, and engaging in interactive conversations in dialogue systems [3, 4]. These capabilities highlight LLMs’ extensive applicability and transformative potential across diverse domains [5].

Once LLMs have been developed for different knowledge domains, a growing need for language models adapted to specific domains [6, 7] and their unique linguistic nuances has emerged, such as BloombergGPT [8] for financial domain, Med-PaLM [9] for medical domain, KAI-GPT^1^ for banking industry, ChatLAW [10] for Chinese legal domain, AD-autoGPT [11] for extracting data from authoritative Alzheimer’s Disease sources, LKAN for clinical staging of liver cancer [12], and Radiology-GPT [13] for various radiology-related applications, including diagnostic assistance, research facilitation, and professional communication. These examples highlight the significant utility of specialized LLMs in real-world, domain-specific applications, particularly in clinical settings. Unlike general-purpose language models trained on massive datasets, specialized language models are fine-tuned or trained on domain-specific data [14], thus allowing to comprehend and generate language models specific to that domain’s unique terminology and linguistic patterns. This specialized approach promises to enhance language models’ accuracy, relevance, and practical application.

However, despite their improved performance, domain-specific LLMs also introduce new challenges, particularly in evaluating the quality and contextual alignment of their outputs. Traditional human evaluation, although reliable, is time-consuming, costly, difficult to scale, and has subjective inconsistencies, especially in highly technical domains where expert validation is required [15].

Additionally, traditional surface-based metrics such as BiLingual Evaluation Understudy (BLEU) [16] and Recall-Oriented Understudy for Gisting Evaluation (ROUGE) [17], while historically valuable for machine translation and summarization, are fundamentally inadequate for medical Q&A evaluation. These metrics rely on n-gram overlap and reward lexical similarity rather than clinical correctness, factual grounding, or contextual safety. As a result, they can assign high scores to fluent but clinically wrong responses, and unfairly penalize semantically accurate answers that are lexically divergent from the reference text. Prior studies have consistently shown that BLEU and ROUGE correlate poorly with expert medical judgments and fail to capture semantic adequacy, reasoning quality, and domain-specific relevance [18]. Because subtle inaccuracies or omitted details can endanger patient safety in hemophilia care, evaluation must focus on clinical correctness, completeness, and reasoning transparency instead of surface similarity alone. Therefore, human-aligned evaluation frameworks and domain-expert assessment are essential to quantify model performance in specialized biomedical Q&A contexts reliably.

To address these shortcomings, alternative methods like GPTScore [19] have been developed, leveraging embeddings and probabilistic outputs from large language models to provide more reliable assessments [19]. Although BERTScore offers a more nuanced, context-aware evaluation by utilizing contextual embeddings, it also presents disadvantages, such as being computationally expensive and sometimes failing to detect smaller errors or repetitions, especially when candidate and reference texts are lexically or stylistically similar. Furthermore, issues like social bias in LLM-based evaluation [20] and the difficulty of assessing low-resource languages remain critical areas of concern [21, 22]. These combined difficulties highlight the pressing need for innovative approaches to optimize the efficiency, reliability, and fairness of LLM development and evaluation.

In this context, this paper presents a comprehensive series of analyses and experiments aimed at evaluating the ability of LLMs to generate accurate and contextually aligned Q&A content within the specialized domain of hemophilia.

Hemophilia is a hereditary disease linked to the X chromosome that affects 1 in every 5000–10,000 male births. Individuals with this condition have mutations, i.e., defective endogenous copies of the Factor VIII (FVIII), Factor IX (FIX), and Factor V genes, which severely affect the blood coagulation process and lead to the development of hemophilia type A, B, and V, respectively. Depending on the mutation, the symptoms of the disease range from mild (few bleeding episodes and coagulation activity around 5–40%) to moderate (more frequent bleeding episodes and coagulation activity between 1–5%) or severe (permanent risk of bleeding, joint issues, chronic joint damage, and coagulation activity below 1%) [23].

Knowledge and treatment of hemophilia lag approximately 15 years behind other health conditions, contributing to significant disparities. Given that treatment costs are prohibitive for 75% of hemophilia patients [24], increased awareness and support from such research could stimulate policy changes and resource reallocation. However, due to its low incidence in the population, hemophilia attracts little interest from specialized medical professionals, researchers, or the pharmaceutical industry. Consequently, there is a notable reduction in the production of materials containing updated information on the disease, its diagnosis, and newly available treatments. The limited number of specialized professionals further exacerbates the clinical workload, hindering the retrieval of materials necessary for ongoing knowledge updates.

Therefore, it is essential to enrich the functionality of an LLM by using specific input text, tailored to the domain of hemophilia, since developing a dedicated Q&A dataset for hemophilia, even if initially generated by a general-purpose LLM, offers a distinct advantage: it enables the identification of model gaps and biases within this specialized domain.

Rather than focusing solely on the generative capabilities of LLMs, our work addresses the critical challenge of evaluating their outputs, particularly within specialized healthcare domains. To this end, we introduce HemoQAL (Hemophilia Question and Answer Learning), a curated Q&A dataset centered on hemophilia, designed to support the evaluation and development of LLMs for specialization domains. In addition, we explore the effectiveness of lightweight and computationally efficient methods for assessing the contextual alignment of LLM-generated content. Specifically, we conduct both human judgment evaluation and semantic similarity analysis over HemoQAL using three well-established text representation: Term Frequency–Inverse Document Frequency (TF-IDF) [25], Word2Vec [26], and Bidirectional Encoder Representations from Transformers (BERT) [27]. They were selected for their objectivity and low computational cost, offering a scalable alternative to traditional human-based or resource-intensive LLM-based evaluation strategies.

The key contributions of this work are summarized in the following points:

- We introduce **HemoQAL**, a curated Q&A dataset for hemophilia built from recent peer-reviewed medical literature and clinical guidelines, ensuring clinically relevant and high-quality content for rare-disease research.
- We explore human judgment analysis to improve the factual accuracy and contextual alignment of content generated by LLMs;
- We explore semantic similarity analysis as a computationally efficient alternative for evaluating the contextual consistency of content generated by LLMs;
- We demonstrate that lightweight semantic similarity metrics, when paired with clinical expert validation, provide a scalable and cost-efficient approach for evaluating medical Q&A data without the computational burden of large-scale model benchmarking.
- By emphasizing methods with low computational cost, our results offer a framework that can be extended to other specialized healthcare domains for applying LLMs in tasks such as decision-making, clinical documentation, and patient interaction.

Based on the preceding discussion, this article is organized as follows: “Background” section presents the foundational concepts underpinning this work and provides a synopsis of how LLMs are increasingly being tailored to address specific domain tasks, along with the assessment of Q&A Generation by LLMs. “HemoQAL: dataset preparation and experimental design” section outlines a Q&A dataset specifically tailored for the domain of hemophilia, while offers a more detailed description of the experiments concerning Human Judgment and Semanctic Similarity Analysis following by a discussion about the overall results in “Discussion” section. “Conclusion” section presents some conclusions, as well as directions for future work.

## Background

This section introduces two foundational concepts to our work: Domain Specialization in LLMs (“Large language models for specialized domains” section), which examines how general-purpose language models often lack the nuanced understanding required for specialized domains; and LLM-based Q&A Generation Assessment (“Assessment of Q&A generation by LLMs” section), which establishes evaluation frameworks for measuring the quality of LLM-generated questions and answers from our medical source. Together, these concepts form the methodological basis for developing and evaluating our hemophilia-specific Q&A dataset.

### Large language models for specialized domains

A language model is a probabilistic model that captures the likelihood of sequences of tokens (such as words or subwords) in text. It predicts the probability of a sequence of tokens based on preceding tokens, enabling it to generate new sequences or predict the next token in a sequence [2]. Consequently, Large Language Models (LLM) constitute foundational models trained on extensive datasets to support diverse applications and tasks across Natural Language Processing (NLP) and Artificial Intelligence (AI) [1]. LLMs, exemplified by OpenAI’s GPTs models,^2^ Meta’s Llama models,^3^ Google’s Gemini,^4^ IBM’s Granite series on watsonx.ai,^5^ and Deepseek,^6^ serve as pivotal generative AI models and frameworks. This innovation allows the generation of human-like text and other content types, getting contextual inference for coherent responses.

The diverse applications of LLMs are achieved through transformer-based architectures, large-scale pre-training, and adherence to scaling laws. Transformer is a deep learning architecture based on the multi-head attention mechanism [28], which computes a weighted sum of input tokens based on their relevance to the current token. This mechanism enables efficient modeling of long text and allows highly parallelized training. The transformer’s scalability and adaptability in focusing on different sequence parts depends on large-scale pre-training, which means a massive corpus of texts is necessary to learn rich linguistic knowledge and language patterns [2]. By increasing the volume of training data, expanding model parameters, and extending the duration of training, LLMs can capture a more comprehensive representation of language patterns, when compared to other architectures, such as Long Short-Term Memory (LSTM) [29].

Although LLMs demonstrate remarkable generalization capabilities and deliver accurate, context-aware responses across broad applications, they still struggle to generate reliable Q&A pairs in highly specialized domains such as healthcare, finance, legal, and scientific research [30, 31]. While fine-tuning on domain-specific data can improve their performance, LLMs often fail to capture the precise nuances, regulatory constraints, and expert-level accuracy required in these fields [32].

Therefore, LLMs for domain specialization is a problem that requires effective techniques to address the challenges associated with it, such as keeping the LLM updated with the latest knowledge, especially in domains where there is a constant production of information such as medicine. According to Zhao et al. [14], domain specialization techniques can be divided into three groups: prompt crafting, external augmentation, and model fine-tuning. Prompt crafting is when a specific input text is utilized to help guide the LLM response generation process and set expectations for the desired output. External augmentation refers to the enhancement of the LLM by retrieving information from external sources, without fine-tuning the model. This can be done by providing the model domain-specific information from an external knowledge source, allowing it to improve its responses, or by integrating the LLM with an external system or tool.

Inspired by these achievements, efforts have been made to tailor general LLMs for the medical domain, leading to the development of specific LLMs [33, 34]. Examples of this type of LLM are MedPaLM [35], MedPrompt [36], ChatDoctor [37], MedAlpaca [38], PMC-LLaMA [39], BenTsao [40], and Clinical Camel [41], resulting in growing research interests in medical LLMs to assisting medical professionals in improving patient care.

Although such LLMs provide good response, broad healthcare datasets generated by then can often do not provide sufficient depth on rare or highly specialized topics, such as hemophilia.

Hemophilia’s rarity leads to limited research attention, but it generates substantial academic output: nearly 6000 publications in just 5 years [42]. This rapid growth of literature makes it increasingly difficult for clinicians, researchers, and students to stay current with the latest findings and clinical guidelines. In this scenario, the development and validation of domain-adapted language models become essential, not only to support automated generation of educational and decision-support materials, but also to ensure that such outputs are reliable, factually accurate, and contextually aligned with the sources. By constructing and evaluating a specialized Q&A dataset for hemophilia, this study contributes to bridging the gap between the wealth of available scientific information and its effective use in ongoing medical education and clinical informatics.

### Assessment of Q&A generation by LLMs

Rapid adoption of LLMs for generating Q&A datasets has brought unprecedented scale and diversity to the creation of resources in specialized domains. Although LLMs can generate responses that often resemble human-produced responses, they still face challenges related to factual accuracy, contextual relevance, and avoidance of bias. Consequently, a systematic evaluation of generated content is essential to ensure the quality, safety, and educational value of AI-powered Q&A systems, especially in sensitive fields such as healthcare [43–45].

In this context, subjective human evaluation remains one of the most widely adopted strategies to validate the quality of LLM-generated Q&A datasets [46]. This approach involves having human reviewers, often domain experts or target users, assess the generated questions and answers based on their personal judgment of correctness, clarity, and relevance. While subjective evaluation captures important qualitative aspects that automated metrics may miss, it is typically performed as a one-time or periodic review, rather than as part of an ongoing, iterative feedback process. This method is especially valuable in domains where nuanced understanding and contextual appropriateness are critical, such as in medical or educational content.

Alongside human evaluation, automatic methods for semantic similarity analysis have been widely adopted to measure the alignment between questions, answers, and reference materials. Techniques such as TF-IDF, Word2Vec, and BERT are used to convert texts into vectors and calculate cosine similarity, enabling a quantitative assessment of how semantically close a generated answer is to the question or original source. The combined use of these methods captures different levels of similarity: from lexical overlap (TF-IDF), through basic semantic relationships (Word2Vec), to deep contextual understanding and linguistic nuance (BERT).

The integration of subjective human assessment and automatic semantic analysis enhances the overall evaluation process for LLM-generated Q&A. While automated methods enable large-scale screening and rapid identification of potentially problematic responses, human review adds contextual judgment, domain expertise, and sensitivity to ethical and pedagogical considerations. This combination is particularly relevant in international benchmarks such as General Language Understanding Evaluation (GLUE), SuperGLUE, Language Modeling Broadened to Account for Discourse Aspects (LAMBADA), and SQuAD (Stanford Question Answering Dataset), which combine objective performance metrics with qualitative evaluations conducted by experts [47].

Recent literature emphasizes that combining subjective human evaluation and semantic similarity analysis raises the standards of quality and trust in Q&A datasets and contributes to the development of models that are better aligned with the real needs of end users [48–50]. This multidimensional approach is regarded as the state of the art for ensuring that LLM-based systems are not only technically advanced but also safe, ethical, and educationally [31, 51, 52]. In the following section, we detail the process used to create the proposed HemoQAL dataset and the experimental setup.

## HemoQAL: dataset preparation and experimental design

This section outlines the experiments using LLM designed to explore the construction and evaluation of our Q&A dataset in the context of hemophilia, referred to as HemoQAL.

Since our work aims to assess the reliability and contextual alignment of Q&A generated by a general-purpose LLM in the hemophilia domain, we address the challenge of evaluating the consistency and relevance of the generated content. To do this, we first created a specialized Q&A dataset based on hemophilia-related texts, followed by two complementary experiments: (i) content validation through a Human Judgment Analysis to ensure quality and educational value; and (ii) semantic similarity analysis by leveraging TF-IDF for keyword overlap, Word2Vec for basic semantic relationships, and BERT for deep contextual understanding.

Such experiments enables a more comprehensive evaluation of alignment between generated questions, answers, and their source materials. This multi-faceted approach enhances dataset reliability by capturing semantic and contextual dimensions that are crucial for high-quality Q&A resources.

### Human judgment analysis

**Fig. 1:**
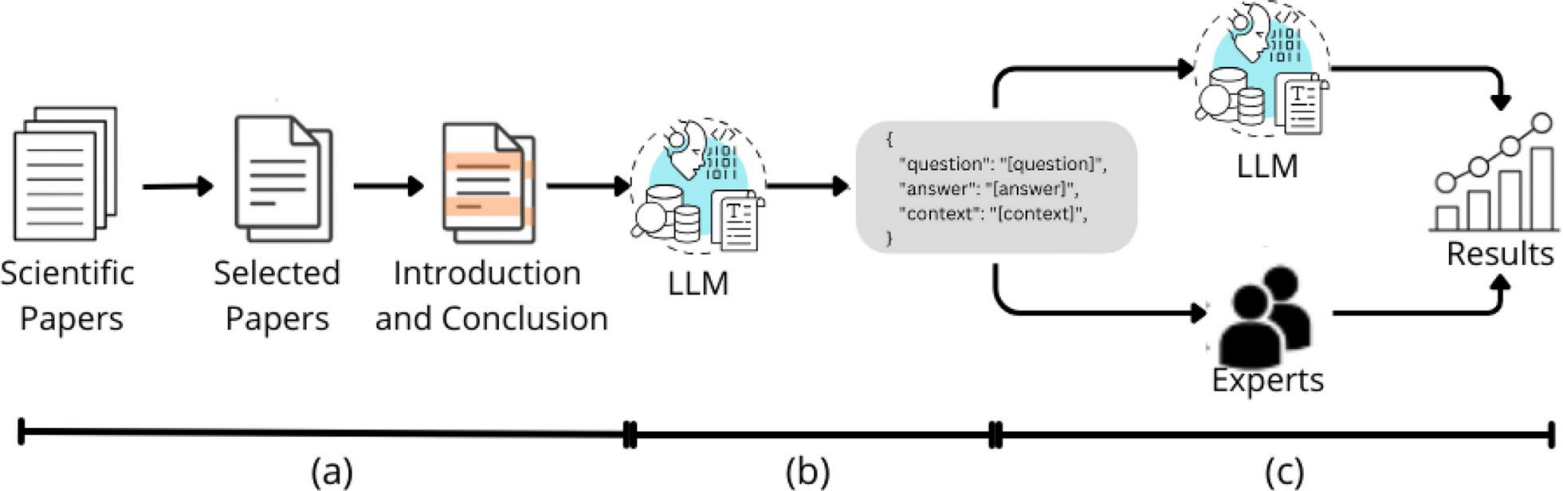
Evaluation of Q&A pair quality through expert judgment in a domain-specific medical dataset

As illustrated in Fig. 1a, we start the construction of our dataset by collecting and selecting the data specific to the hemophilia domain. Initially, we comprehensively reviewed 20,000 papers on hemophilia sourced from various scientific databases. As an initial empirical analysis designed to in-depth assess our proposal, we selected 92 papers from this extensive pool of information that met specific criteria: papers published between 2015 and 2022, papers available with a permissive license on PubMed Central, and papers containing relevant and high-quality information about hemophilia. Next, the “Introduction” and “Conclusion” sections of the selected papers were manually parsed to extract critical information concerning their subjects regarding disease management, treatment options, clinical trials, research findings, and patient support resources.

In the next step, Fig. 1b, the extracted data was used by LLM^7^ to create question-and-answer pairs, inspired by the methodology described by Finardi et al. [53]. Creating these pairs involved carefully formulating questions to ensure they were representative of the content and context provided in the articles, and the subsequent generation of precise and relevant answers by the model. The prompt used to generate the pairs was designed to produce a dataset similar to Stanford Question Answering Dataset (SQuAD^8^) [54], meaning each question was answered with content found in the “Introduction” or “Conclusion” sections of the respective paper.

Although the generation phase relied on a single LLM instance, the resulting Q&A pairs were subsequently evaluated using 13 LLMs to strengthen robustness and ensure cross-model consistency. Moreover, the primary objective of this study is not to benchmark large-scale generative performance across models, but rather to introduce a clinically grounded, human-validated dataset and demonstrate the feasibility and importance of rigorous human-in-the-loop evaluation in this domain. Generating multiple Q&A versions using several LLMs would not strengthen the proposed contribution; instead, it would complicate and dilute the clinical evaluation, reducing clarity and human-assessment rigor, while substantially increasing cost and annotation burden.

Consider an example of a question-and-answer pair generated by LLM from the hemophilia-related papers:
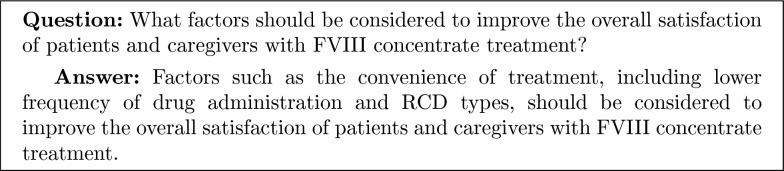


Finally, in the last step, Fig. 1c, the pairs of question-and-answer obtained in the previous step were evaluated by both LLMs and domain experts. An informed consent was obtained from all participants involved in this phase of this study, in accordance with ethical research guidelines.

The human judgment analysis of question-and-answer pairs was conducted in two stages. In the first stage, each generated question was assessed using a categorical scoring system based on the following criteria:

- **0** The question does not make sense or is poorly formulated;
- **1** The question makes sense but looks focused on a specific subject of the paper;
- **2** The question makes sense and can be answered by an expert, regardless of the specific subject of the paper;
- **3** The question is outside the expert’s knowledge.

Considering that the primary goal of our proposal is to generate questions that can be answered by domain experts regardless of the specific subject of the paper, any question that receives a score of 2 from experts is further evaluated in a second stage focused on answer quality.

In this context, we aim to ensure that generated answers are not only correct, but also sufficiently informative and appropriately scoped. To achieve this, we defined three complementary evaluation criteria:

- **Correct answer** Does the answer provide factually accurate information that directly addresses the question? (Yes or No)
- **Complete answer** Does the answer include all relevant information that a domain expert would reasonably expect, beyond the minimal correct response (e.g., missing clinical details, conditions, or contextual nuances)? (Yes or No)
- **Concise answer** Does the answer provide only the necessary information required to address the question, without including irrelevant or excessive details? (Yes or No)

These criteria were explicitly defined to evaluators to distinguish factual validity (correctness) from informational sufficiency (completeness) and response scope (conciseness).

These assessment questions are not exclusive, and this evaluation was provided by thirteen LLMs and four domain experts (bioinformatics specializing in hematology). Their evaluation was analyzed, and the results are discussed as following.

Considering the two-stage evaluation protocol previously described, Table 1 presents the results of the first stage of the human-judgment analysis. This stage focused specifically on question quality, applying the four-level categorical scoring framework illustrated in Fig. 1.

The distribution of scores reveals heterogeneous behavior among evaluators. Human experts demonstrated distinct evaluation profiles: Expert 1 exhibited a balanced distribution across all score categories, suggesting a nuanced and calibrated judgment of question clarity and relevance. Expert 2 adopted a comparatively conservative stance, assigning more scores in categories 0 and 1, indicative of strict filtering criteria regarding clinical significance and generalization. Meanwhile, Experts 3 and 4 assigned a larger proportion of questions to categories 2 and 3, indicating that many questions were deemed answerable by clinicians or, in some cases, extended beyond their specific expertise.

By contrast, LLM-based evaluators displayed a markedly different distribution. Several models, such as DeepSeek-chat and Gemini-2.5-flash-lite, concentrated the majority of scores in category 2, reflecting high confidence in the coherence and clinical validity of the questions. GPT-based models, particularly GPT-4.1 and GPT-4o, similarly assigned most items to category 2, consistent with the strong performance expected from state-of-the-art models. Notably, however, LLMs rarely selected category 3, in contrast with human experts, who recognized domain boundaries explicitly. This pattern suggests that while advanced LLMs are highly capable of recognizing clinically meaningful questions, they may underestimate knowledge uncertainty, potentially overextending their confidence.

Overall, the first-stage results highlight complementary tendencies: human evaluators emphasize domain specificity and correctness, whereas LLMs present more permissive and optimistic assessments of question validity. These differences reinforce the importance of combining automated evaluation with domain-expert oversight in medically sensitive settings. Such findings align with well-documented limitations of LLMs in clinical applications, particularly their propensity toward hallucination and overconfidence [55], underscoring the continued need for human supervision in the development and validation of medical Q&A resources.

**Table 1:** Results obtained using the four-level categorical scoring framework across experts and LLMs

Evaluators	0	1	2	3
Expert 1	3	14	48	26
Expert 2	6	23	62	0
Expert 3	2	14	59	16
Expert 4	6	15	66	4
Deepseek-chat	1	12	78	0
Deepseek-reasoner	5	4	79	3
Gemini-2.0-flash	3	17	70	1
Gemini-2.0-flash-lite	2	16	73	0
Gemini-2.5-flash	0	16	71	4
Gemini-2.5-flash-lite	2	9	80	0
Gemini-2.5-pro	1	14	71	5
GPT-4.1	0	9	81	1
GPT-4o	1	18	70	2
GPT-4o-mini	1	16	70	4
GPT-5	5	15	70	1
GPT-o3	0	9	81	1
Llama	0	13	76	2

**Fig. 2:**
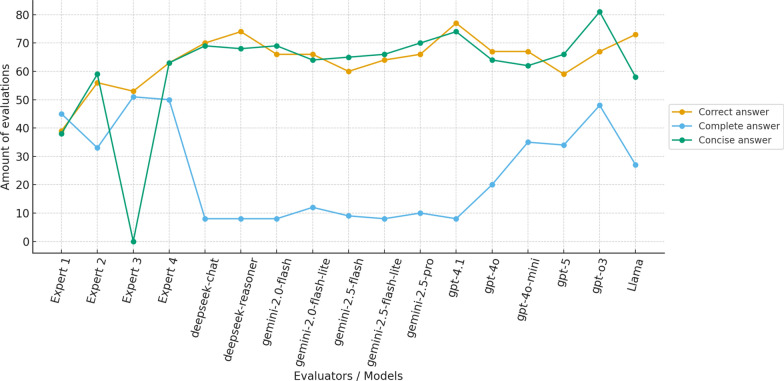
Distribution of evaluation outcomes across human experts and LLMs for three assessment dimensions: Correct answer, Complete answer, and Concise answer. Values represent the number of evaluations assigned to each category per evaluator

Subsequently, in the second stage of our evaluation, for each question receiving a score of 2, thirteen LLMs and four domain experts produced answers, and their performance is summarized in Fig. 2. The results reveal clear behavioral differences between human experts and LLMs across the three dimensions analyzed (Correct answer, Complete answer, and Concise answer). Human experts demonstrate a more heterogeneous evaluation profile, with balanced distributions and stricter assessments regarding correctness and completeness. In particular, Expert 2 maintains relatively conservative judgments, while Experts 3 and 4 tend toward higher correctness and conciseness scores.

In contrast, LLMs exhibit consistently high scores for correctness and conciseness, often surpassing human evaluators in these categories. However, they show considerably lower completeness values, suggesting that while LLM answers are generally accurate and concise, they may omit clinically relevant detail–reflecting a trade-off between brevity and depth. This pattern is consistent with the confidence observed in Table 1, where LLMs tended to classify most questions as valid and answerable.

Overall, these findings highlight complementary strengths: LLMs provide consistent and concise outputs, whereas domain experts offer more nuanced and completeness-oriented evaluations–underscoring the necessity of human oversight in sensitive medical domains and reinforcing the value of a hybrid evaluation framework.

To illustrate the patterns observed in the broader second-stage evaluation (Fig. 2), Table 2 presents a small subset of Q&A pairs that received a score of 2 during the first stage. From the full set of four experts and multiple LLMs, two domain experts (Expert 1 and Expert 2) and the same model used to generate the original dataset (GPT-4o) were selected for this qualitative analysis. The **ID** column identifies each example, while the **Correct**, **Complete**, and **Concise** columns report the evaluations given by these selected annotators.

This illustrative subset highlights key disagreement patterns. For instance, in Example 1, GPT-4o classifies the answer as concise, whereas both experts disagree. Conversely, in Example 4, Expert 2 judges the answer to be concise, while GPT-4o does not. These cases suggest that the model may be less confident when responses contain more nuanced or elaborated clinical detail. Additionally, in Example 2, both experts consider the answer complete, whereas GPT-4o does not, illustrating the broader trend observed in Fig. 2 where LLMs tend to provide lower completeness scores. Notably, in these cases, both experts and GPT-4o consistently agree on correctness, indicating that divergences are mainly related to completeness and conciseness judgments.

Example 3 demonstrates the opposite scenario: GPT-4o classifies the answer as correct, while both experts disagree. This indicates that as questions require deeper domain knowledge or more context, the model’s confidence does not always translate into clinical accuracy, underscoring the need for human oversight in specialized medical settings.

**Fig. 3:**
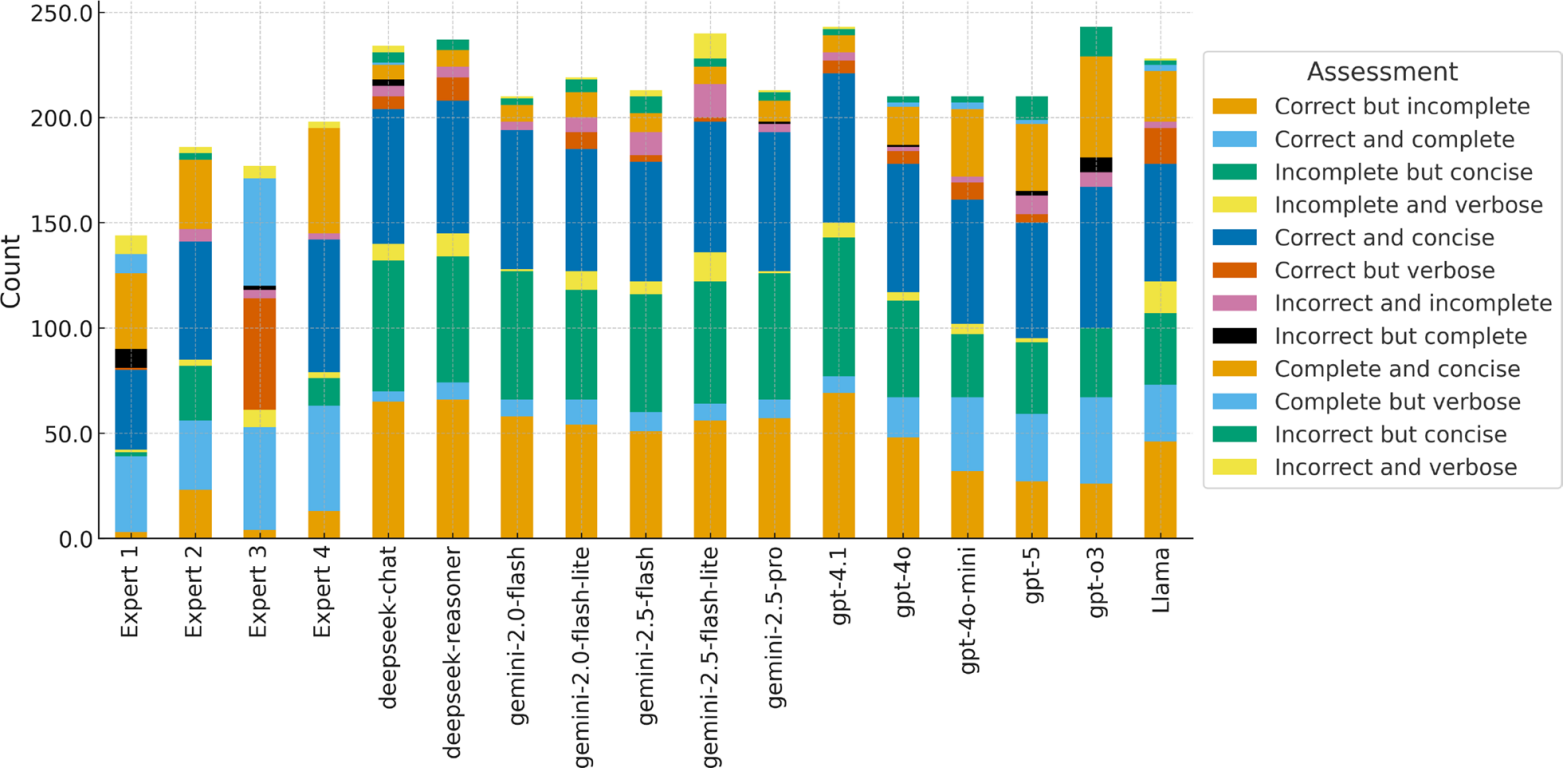
Distribution of assessment outcomes assigned by four domain experts and thirteen LLMs across combined correctness, completeness, and conciseness categories (e.g., Correct but incomplete, Complete and concise, Incorrect and verbose). The stacked bars illustrate how experts apply more varied judgments, while LLMs tend to concentrate evaluations in correct and concise categories, with fewer incomplete or incorrect cases flagged

A summary of the quantitative results for all evaluators is shown in Fig. 3, comparing expert and model performance across correctness, completeness, and conciseness.

**Table 2:** Examples of Q&A pairs with different evaluations by human experts and the LLM (GPT-4o)

ID	Q & A	Correct	Complete	Concise
1	**Q:** What can cause false low activity values for FVIII and FIX?			
	**A:** Rivaroxaban causes false low activity values for FVIII and FIX	No/Yes/Yes	No/No/Yes	No/ No/Yes
2	**Q:** What is surface electromyography used for in patients with haemophilia?			
	**A:** Surface electromyography is used to assess muscle function in patients with haemophilia	Yes/Yes/Yes	Yes/Yes/No	No/Yes/Yes
3	**Q:** What is one likely outcome for gene therapy in hemophilia?			
	**A:** Gene therapy for hemophilia will not yet mean the end of phenotypic testing	No/No/Yes	Yes/No/Yes	No/No/Yes
4	**Q:** What is the general outcome of post-operative treatment in haemophilic knee arthropathy?			
	**A:** The post-operative outcome in haemophilic knee arthropathy may not be as good as in regular cases, but there is significant improvement in functional outcome and quality of life	Yes/Yes/Yes	No/No/Yes	No/Yes/No

The evaluation pattern *x/x/x* represents scores assigned by *Expert 1/ Expert 2/ LLM*, respectively, for each criterion (Correct, Complete, Concise)

Figure 3 provides a detailed breakdown of assessment outcomes across expert reviewers and LLMs, combining correctness, completeness, and conciseness judgments into compound categories (e.g., Correct but incomplete, Complete and concise, Incorrect and verbose). A clear contrast emerges between human evaluators and LLMs.

Human experts display a broader distribution of judgments, indicating more nuanced and variable assessments. For instance, Experts 1 and 2 allocate a considerable proportion of responses to Correct but incomplete and Correct and complete, but also register notable frequencies of Incomplete but concise and even Incorrect and incomplete assignments. This variability suggests a stricter and context-sensitive evaluation, likely influenced by their clinical perspective and awareness of subtle domain-specific nuances. Expert 3 shows more dispersion across some categories, reflecting a more critical stance, whereas Expert 4 leans toward correctly identifying concise and complete answers but still diverges meaningfully from LLM patterns.

In contrast, LLMs tend to cluster around a narrower set of positive categories. Models such as GPT-4.1, GPT-4o, and Gemini variants show a high concentration of Correct and concise and Correct but incomplete evaluations, reinforcing the trend previously observed that LLMs often generate correct answers but may omit clinically meaningful depth. Additionally, LLMs present fewer instances of Incorrect categories, suggesting either greater consistency or, potentially, a systematic bias toward optimistic self-assessment when judging their own or another model’s responses. Some models (e.g., GPT-o3 and DeepSeek-reasoner) also display relatively strong completeness profiles, whereas others (e.g., smaller Gemini variants) show greater dispersion combining conciseness with incomplete reasoning.

To assess the inter-rater reliability among the human experts, we calculated Fleiss’ Kappa ($\kappa$) for each evaluation criterion across all instances (questions). The panel demonstrated a reliable baseline for objective evaluation, achieving a fair-to-moderate agreement for factual correctness ($\kappa = 0.305$). As anticipated for highly subjective and stylistic metrics, the agreement was lower for completeness ($\kappa = 0.057$) and conciseness ($\kappa = 0.048$).

Taken together, these results highlight complementary strengths and limitations: LLMs produce coherent and concise judgments at scale, whereas human experts provide deeper scrutiny, particularly concerning completeness and context relevance. This divergence again underscores the necessity of hybrid evaluation frameworks in safety-critical medical domains, where completeness and clinical nuance are essential for reliable knowledge assessment.

Finally, it is important to note that, although human expert involvement is crucial for validating and interpreting LLM performance in rare-disease contexts such as hemophilia, recruiting a sufficient number of qualified medical reviewers is substantially challenging and resource-intensive. This constraint reinforces the value of LLM-assisted evaluation pipelines, while simultaneously emphasizing that expert-in-the-loop processes remain indispensable.

Therefore, the main contribution of this work is a modular framework that allows consistent use of human judgment in different fields through standardized evaluation, making it adaptable to various LLMs while keeping the core comparison between human and LLM.

In addition to our proposal of using human judgment analysis for HemoQAL, we have also conducted a semantic similarity analysis in order to quantitatively assess the alignment between the model-generated responses and our source. This complementary approach helps to evaluate how closely the model’s outputs match the intended meaning of hemophilia domain, providing an additional layer of validation that supports or contrasts the qualitative results obtained through human evaluation.

### Semantic similarity analysis

**Fig. 4:**
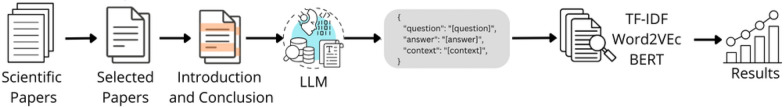
Semantic similarity evaluation for Q&A content in a specialized clinical domain

Our semantic similarity analysis was conducted using the pipeline illustrated in Fig. 4. After constructing HemoQAL as described earlier, we processed pairs of texts through TF-IDF, Word2Vec, and BERT embeddings to assess their semantic alignment and contextual relevance. For the HemoQAL dataset, this involved evaluating the correspondence between: questions and their answers, LLM-generated contexts and the original source texts, as described in Table 3.

When constructing the HemoQAL dataset, GPT-4o was used to generate the question–answer pairs from the selected medical articles. During this process, GPT-4o also produced an auxiliary context, a synthesized segment of text that the model relied on to structure its reasoning and ensure that the generated questions and answers remained coherent, relevant, and faithful to the original source material. This context consists of both the provided material, in our case the documents about hemophilia, and the model’s pre-trained knowledge, allowing it to formulate questions and responses while maintaining logical consistency. The supplied material anchors the content, while the LLM’s internal knowledge fills gaps, enhances explanations, or adapts complexity based on the task. However, if the material used is too limited, the model might guess too much based on what it learned before, which can lead to incorrect answers. Thus, the LLM-generated context is a mix of what the user provides and what the model has learned.

Therefore, the pairwise comparisons shown in Table 3 evaluate the semantic consistency between the elements generated by GPT-4o for the HemoQAL dataset and the original source documents. These comparisons capture alignment at multiple linguistic levels, ranging from lexical similarity (TF-IDF) to deeper contextual representation (BERT-based embeddings), ensuring that GPT-4o’s outputs remain grounded in the source text.

**Table 3:** Pairwise consistency checks between questions, answers, context, and source documents

Comparison	Objective
Question $\leftrightarrow$ LLM context	Verifies whether the generated context is relevant and coherent with the original question
Answer $\leftrightarrow$ LLM context	Assesses whether the answer is appropriately supported by the provided context
LLM Context $\leftrightarrow$ Original document	Evaluates the factual alignment between the generated context and the original source material
Question $\leftrightarrow$ Original document	Checks whether the original document contains sufficient information to support the question
Answer $\leftrightarrow$ Original document	Measures the faithfulness of the generated answer to the original source content

The evaluation concerning text representation of the Q&A dataset obtained began with the vectorization of all elements of the HemoQAL dataset, starting by a preprocessing step to clean the data by removing stopwords, punctuation, and other irrelevant tokens and the training was made with Google’s model. After cleaning, words were represented as continuous vectors in a semantic space, capturing their relationships based on co-occurrence patterns. Semantic alignment between text pairs was then evaluated using cosine similarity [56], which is a metric that measures the angle between two vectors in a multi-dimensional space, providing a value between $-1$ and 1, where higher values indicate greater similarity. Formally, given two vectors $\textbf{A}$ and $\textbf{B}$, their cosine similarity is defined as:

$$\text {cosine}(\textbf{A}, \textbf{B}) = \frac{\textbf{A} \cdot \textbf{B}}{\Vert \textbf{A}\Vert \cdot \Vert \textbf{B}\Vert } = \frac{\sum _{i=1}^{n} A_i B_i}{\sqrt{\sum _{i=1}^{n} A_i^2} \cdot \sqrt{\sum _{i=1}^{n} B_i^2}},$$

where $\textbf{A} \cdot \textbf{B}$ is the dot product of vectors $\textbf{A}$ and $\textbf{B}$, and $\Vert \textbf{A}\Vert$ and $\Vert \textbf{B}\Vert$ are the Euclidean norms (magnitudes) of the vectors. A result equal to **1** indicates identical orientation (maximum similarity), **0** denotes orthogonality (no correlation), and **– 1** represents diametrical opposition (maximum dissimilarity).

For this purpose, our dataset was converted into three vector representations, which were then compared using cosine similarity.

Firstly, it was created a Bag of Words (BoW), which is composed by sparse vectors to represent word frequency distributions for each element in the datasets. This method focused on identifying lexical overlap and comparing word frequency across text pairs, such as titles and comments or questions and contexts. By highlighting direct word-level matches, BoW provided a straightforward measure of textual alignment. Term Frequency–Inverse Document Frequency (TF-IDF) [25] extended the analysis by emphasizing the significance of less frequent, contextually important words in the texts. By calculating a weighted score for each term, TF-IDF highlighted words that were particularly representative of the content, such as unique terms in questions or comments. This allowed for a more detailed evaluation of information specificity and its relevance to the associated context.

Next, Word2Vec [26] was used to encode the words as dense vectors in a high-dimensional space. The proximity between these vectors reflects the semantic similarity between the corresponding words, meaning that words with similar contexts tend to be located close to each other in this space. This vector representation enables computers to capture not only basic relationships like synonymy but also more complex associations such as analogies. As a result, Word2Vec provides a powerful foundation for understanding and modeling language in a way that aligns more closely with human-like interpretation.

Finally, Bidirectional Encoder Representations from Transformers (BERT) [27] was used for deeper semantic comparisons across our dataset. Leveraging its advanced understanding of sentence-level and paragraph-level context, BERT produced dense embeddings that captured nuanced semantic relationships between text pairs. These embeddings enabled precise evaluations of coherence and alignment, revealing subtle connections between questions and contexts across HemoQAL.

**Fig. 5:**
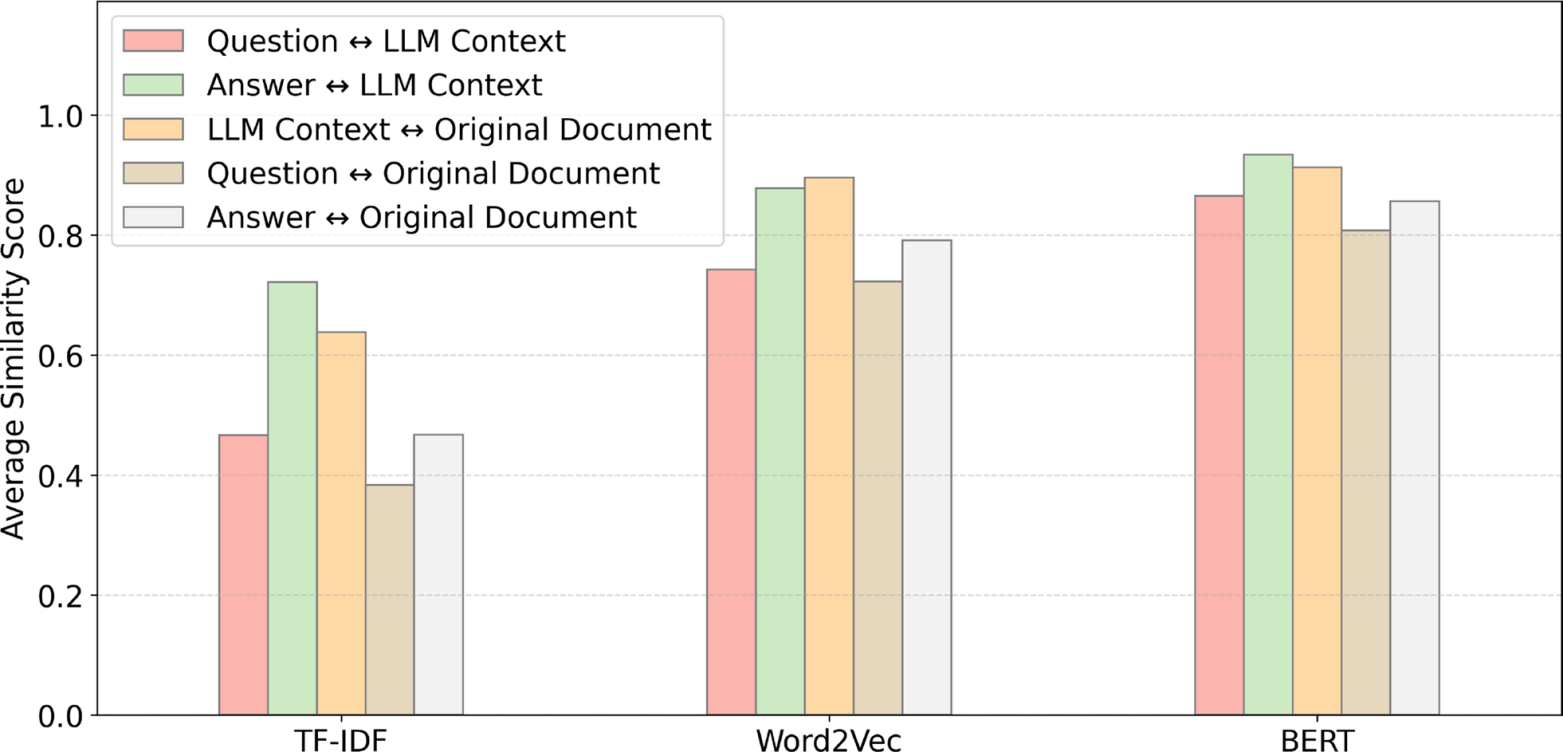
Average similarity score for the hemophilia dataset

In summary, our evaluation aimed to assess coherence, relevance, and specificity, leveraging semantic similarity as a key indicator of contextual alignment, as displayed in Fig. 5, and discussed next. The data in the figure is visually organized using colors to represent different comparison pairs while the groups are divided by type of text representation, providing a clear and structured visualization of the results.

Onto an analysis of the suitability of the questions, answers, and contexts generated by LLM, presented in Fig. 5, the results from the methods studied provide informations into their alignment with the original documents. High similarity scores achieved by methods like BERT and Word2Vec suggest that the generated content is semantically consistent with the source material. This indicates that, for the most part, the questions and answers produced are contextually grounded in the original documents.

However, the differences in performance across methods highlight varying levels of reliability. While BERT demonstrates a strong and nuanced ability to capture contextual alignment, methods like TF-IDF yield noticeably weaker scores, reflecting limitations in their capacity to capture deeper lexical and semantic relationships within the generated content.

Focusing on the similarity between the LLM-generated context and the original documents, the results indicate a high similarity scores, consistently above 0.6 score, which suggests a strong alignment between the generated contexts and the source documents. This implies that the generated contexts likely preserve the semantic and informational content of the original texts, supporting the inference that they are within the same context. While Word2Vec demonstrates strong similarity scores across various comparison pairs, it does not outperform BERT, only coming close in the context to original documents metric, which is expected, since they supposedly have more context in common.

When comparing the questions and answers with the LLM-generated context and the original documents, the results provide additional results into the quality and alignment of the generated content. The similarity scores indicate a stronger alignment between the questions and answers and the LLM-generated context, which is expected given that the generated context is significantly shorter and more focused compared to the original documents. This conciseness reduces semantic variability, making it easier for the questions and answers to maintain a high level of similarity.

Additionally, it is notable that the similarity scores for answers, when compared to both the LLM-generated context and the original documents, are consistently higher than those for questions. This trend is logical, as answers typically contain more direct information aligned with the context or the document, reflecting explicit content that is easier to match semantically. In contrast, questions often introduce more variability due to their interrogative structure, which may not always align as closely with the phrasing or content of the source material.

Our semantic similarity analysis across different text representation reveals clear distinctions in their effectiveness for capturing contextual alignment. BERT consistently outperforms other models, demonstrating robust performance across all datasets, including structured scientific texts in the Hemophilia dataset. Its ability to capture nuanced semantic relationships at both sentence and paragraph levels makes it a reliable tool for evaluating the contextual alignment of generated content with source materials.

Word2Vec, while moderately effective, particularly in structured datasets, falls short in handling more complex and varied text. In contrast, TF-IDF, which rely on lexical overlap rather than deep semantic understanding, consistently underperformed, demonstrating their limitations in tasks requiring contextual awareness.

In contrast, simpler method like TF-IDF consistently underperform, with low similarity scores. Their reliance on lexical overlap and word frequency limits their ability to capture deeper semantic relationships, especially in complex, domain-specific, or conversational contexts. Despite their computational efficiency, these methods are less suitable for tasks that require fine-grained semantic evaluation, particularly when assessing the coherence of LLM-generated content.

These patterns suggest that the LLM-generated context effectively acts as a semantic bridge, simplifying and concentrating key information from the original documents, thus facilitating a closer alignment with the crafted questions and answers. The stronger alignment of answers further reinforces the coherence between the generated content and the source material.

## Discussion

This study introduces a novel evaluation approach to create a comprehensive Q&A dataset. This approach uses advanced AI techniques to produce questions and answers that are contextually appropriate for specialized domains. Our approach incorporates a Human Judgment Analysis framework, which integrates AI capabilities with evaluation from human experts. This collaborative model ensures rigorous validation of the generated Q&A dataset, maintaining standards of relevance within a specialized domain. The most important contribution of this work is the publication of our complete dataset, comprising pre-processed manuscripts along with Q&A from LLM and specialists. Our dataset enables researchers to design new LLMs based on our texts and use the Q&A information as benchmarks. As future work, the resultant Q&A dataset serves as a foundational resource for developing an interactive educational platform. This platform is envisioned to continually expand its content with informative articles, educational presentations, and clinical guides. Our work seeks to enhance educational opportunities in specialized domains, facilitating broader access to up-to-date knowledge.

In addition, the results concerning the semantic analisys provide evidence regarding the context alignment capabilities of the evaluated methods, particularly BERT. The consistent high performance of BERT suggests that it is highly effective at capturing semantic relationships. This indicates that BERT can reliably identify correlations between texts even when dealing with specialized content.

As a result, by consolidating a specialized repository of hemophilia-related questions and answers, our evaluation aims to improve the precision, relevance, and practical application of medical information in this domain. This initiative supports open-access publishing and data sharing and accelerates advancements in hemophilia and rare disease research.

Dataset-specific trends indicate that structured, domain-specific texts, such as the Hemophilia Q&A dataset, allow for slightly better performance of simpler methods, but as text complexity increases, only advanced contextual models like BERT maintain reliability. Additionally, the higher similarity scores for generated answers compared to questions suggest that while LLM-generated responses maintain coherence, extra validation might be necessary for questions to ensure alignment with source content.

Besides, the inter-rater reliability results offer important insights into the nature of expert evaluation within this domain. The fair-to-moderate agreement observed for factual correctness validates the reliability of our expert panel, demonstrating a stable, shared baseline when assessing the core accuracy of the generated answers. In contrast, the sharp drop in agreement regarding completeness and conciseness should not be interpreted as random annotation noise. Rather, it empirically reflects the inherent subjectivity of these criteria. Experts with varying practical workflows naturally have different thresholds for what constitutes sufficient or excessive detail. This divergence strongly supports our interpretability-focused evaluation strategy, highlighting that while domain experts can reliably align on objective facts, their stylistic preferences and operational requirements are fundamentally diverse.

Overall, these findings demonstrate that BERT is the only method that consistently delivers promising results in evaluating the quality and contextual alignment in LLM-generated content. However, its consistently high similarity scores should be interpreted with caution, as they may indicate a tendency to overgeneralize rather than deeply capture contextual nuances. Future studies should investigate this further by incorporating qualitative analysis or human-in-the-loop evaluations to verify whether BERT’s assessments truly reflect contextual fidelity.

We also note that the present analysis is based on aggregated similarity scores across different embedding methods and comparison settings, which allows us to identify consistent trends but does not capture relationships at the individual instance level. As such, while the observed patterns suggest that semantic similarity measures reflect meaningful aspects of answer quality, they should be interpreted as complementary indicators rather than direct proxies for expert evaluation. A more rigorous assessment of alignment between automated metrics and human judgments would require instance-level analyses, such as correlation, agreement, or predictive modeling, which we leave as an important direction for future work.

## Conclusion

In conclusion, this study highlights the value of combining human judgment with computational semantic similarity metrics for assessing contextual alignment of Q&A dataset. Our approach provides an alternative to traditional methodologies that rely exclusively on either manual analysis or automated scoring, enabling more nuanced and scalable evaluations of textual correspondence.

Notably, the stronger alignment of LLM-generated answers compared to questions highlights the inherent differences in how each aligns with source content. Answers, often more explicit and context-bound, exhibit higher semantic similarity, while questions introduce variability due to their interrogative structure. This suggests that while LLM-generated content maintains strong coherence, additional validation may be required for generated questions to ensure precise contextual alignment.

Overall, our analysis demonstrates that combining expert validation with modern embedding-based similarity metrics provides a reliable mechanism for assessing factual consistency and semantic grounding in domain-specific LLM outputs.

To complement this analysis, integrating advanced evaluation frameworks such as RAGAS [57] and DEEPEVAL [58] presents a compelling direction for future work. However, these frameworks require higher computational resources for leveraging sophisticated evaluation techniques, including refined semantic and contextual alignment metrics. Their incorporation into automated evaluation pipelines could help address potential limitations observed in BERT, ensuring a more nuanced and scalable approach to assessing LLM-generated content. Future research should explore these frameworks across diverse domains to systematically evaluate the comparative strengths and limitations of different assessment approaches. Such investigations would enable the refinement and advancement of LLM evaluation methodologies. Our dataset remains publicly available to support these research efforts and facilitate reproducible comparisons.

## Data Availability

The datasets and code used in this study are available at https://github.com/LabIA-UFBA/HemoQAL.

## Footnotes

1 https://kasisto.com/products/kai-gpt/

2 https://platform.openai.com/docs/models.

3 https://llama.meta.com/.

4 https://ai.google/.

5 https://www.ibm.com/blog/building-ai-for-business-ibms-granite-foundation-models/.

6 https://www.deepseek.com/.

7 version GPT-4o-2024-05-13.

8 https://rajpurkar.github.io/SQuAD-explorer/.
